# Preliminary impact of an mHealth education and social support intervention on maternal health knowledge and outcomes among postpartum mothers in Punjab, India

**DOI:** 10.1186/s12884-025-07310-y

**Published:** 2025-03-05

**Authors:** Alison M. El Ayadi, Nadia G. Diamond-Smith, Mona Duggal, Pushpendra Singh, Preetika Sharma, Jasmeet Kaur, Lakshmi Gopalakrishnan, Navneet Gill, Garima Singh Verma, Alka Ahuja, Vijay Kumar, Laura Weil, Rashmi Bagga

**Affiliations:** 1 University of California, San Francisco, San Francisco, USA; 2 Postgraduate Institute of Medical Education & Research, Chandigarh, India; 3 Indraprastha Institute of Information Technology Delhi, New Delhi, India; 4 Survival of Women and Children Foundation, Panchkula, India

**Keywords:** Digital health, Social support, Maternal morbidity, Health-care seeking behavior, Health literacy

## Abstract

**Background:**

Significant disruptions in the perinatal continuum of care occur postpartum in India, despite it being a critical time to optimize maternal health and wellbeing. Group-oriented mHealth approaches may help mitigate the impact of limited access to care and the lack of social support that characterize this period. Our team developed and pilot tested a provider-moderated group intervention to increase education, communication with providers, to refer participants to in-person care, and to connect them with a virtual social support group of other mothers with similarly aged infants through weekly calls and text chat.

**Methods:**

We analyzed the preliminary effectiveness of the pilot intervention on maternal health knowledge through 6 months postpartum among 135 participants in Punjab, India who responded to baseline and endline surveys. We described change in knowledge of maternal danger signs, birth preparedness, postpartum care use, postpartum physical and mental health, and family planning use over time between individuals in group call (synchronous), other intervention (asynchronous), and control groups.

**Results:**

Participant knowledge regarding danger signs was low overall regarding pregnancy, childbirth and the postpartum period (mean range of 1.13 to 2.05 at baseline and 0.79 to 2.10 at endline). Synchronous participants had a significantly higher increase over time in knowledge of danger signs than asynchronous and control group participants. Birth preparedness knowledge ranged from mean 0.89–1.20 at baseline to 1.31–2.07 at follow-up, with synchronous participants having significantly greater increases in comparison to the control group. Synchronous participants had nearly three-fold increased odds of postpartum health check with a clinical provider than asynchronous participants (OR 2.88, 95% CI 1.07–7.74). No differences were noted in postpartum depressive and anxiety symptoms.

**Conclusions:**

Preliminary effectiveness results are promising, yet further robust testing of the MeSSSSage intervention effectiveness is needed. Further development of strategies to support health knowledge and behaviors and overcoming barriers to postpartum care access can improve maternal health among this population.

**Supplementary Information:**

The online version contains supplementary material available at 10.1186/s12884-025-07310-y.

## Background

Ensuring access to high-quality maternal health care across each stage of the perinatal continuum of care is critical for reducing maternal morbidity and mortality and improving pregnancy and postpartum experiences. India has made dramatic progress in reducing maternal mortality over the past three decades with an 81% reduction from 556 per 100,000 live births in 1990 to 103 per 100,000 live births in 2017–2019 [1–3]. Supported by a series of Indian-government-initiated maternal and neonatal health schemes [4–6], including an extensive community health workers program [7–9], national data illustrate continued improvement in access to key reproductive health and perinatal care points, with current estimates as follows: early antenatal care receipt (70.0%), a minimum of four antenatal care visits (58.1%), institutional births (88.6%), maternal and child postnatal check-up by skilled health personnel within two days of childbirth (78.0% and 79.1%, respectively) and decreases in unmet need for contraception (9%) [10]. While basic measures are up, indicators of high-quality care, such as maternal consumption of iron and folic acid (26.0% for 180 days or more), anemia among pregnant women (52.2%), and postnatal checks beyond the first week of birth remain below recommendations [10]. Additionally, notable deficits persist in equity across socioeconomic status and rurality [11]. Thus, intensified efforts are required to address quality and equity.

Significant disruptions in the perinatal care continuum occur postpartum, where limited care is provided beyond the first two days after childbirth. High-quality postpartum care and social support has been associated with reductions in maternal and neonatal mortality [12, 13], and increased engagement throughout the postpartum period in behaviors promoting maternal (e.g., appropriate care-seeking for maternal postpartum health concerns, postpartum contraceptive adoption) and newborn health (e.g., exclusive breastfeeding and vaccination acceptance) [14, 15].

Innovations in postpartum care provision and linkage are needed to overcome persisting barriers to postpartum care access and improve postnatal health knowledge and evidence-based behaviors. Prevalent barriers include poverty, low education, social norms around health-related decision-making, gender norms around mobility, lack of health insurance, costs, and perceptions of poor quality or lack of benefit of services [16–18]. Group-oriented mHealth (or digital health) approaches may help mitigate the dual impact of the limited care access and lack of social support that characterize this period. Furthermore, group mHealth approaches are promising logistically, particularly given current high mobile phone penetration and low cost. Their potential builds on the evidence bases of group participatory learning and action models and mHealth, including interventions which have successfully increased antenatal care, birth planning, breastfeeding, infant vaccination, and perinatal social support for improved mental health [19–27], improved health and social support outcomes when applied to other health topics [28, 29], and acceptability and costing of mHealth interventions [30, 31].

**Fig. 1:**
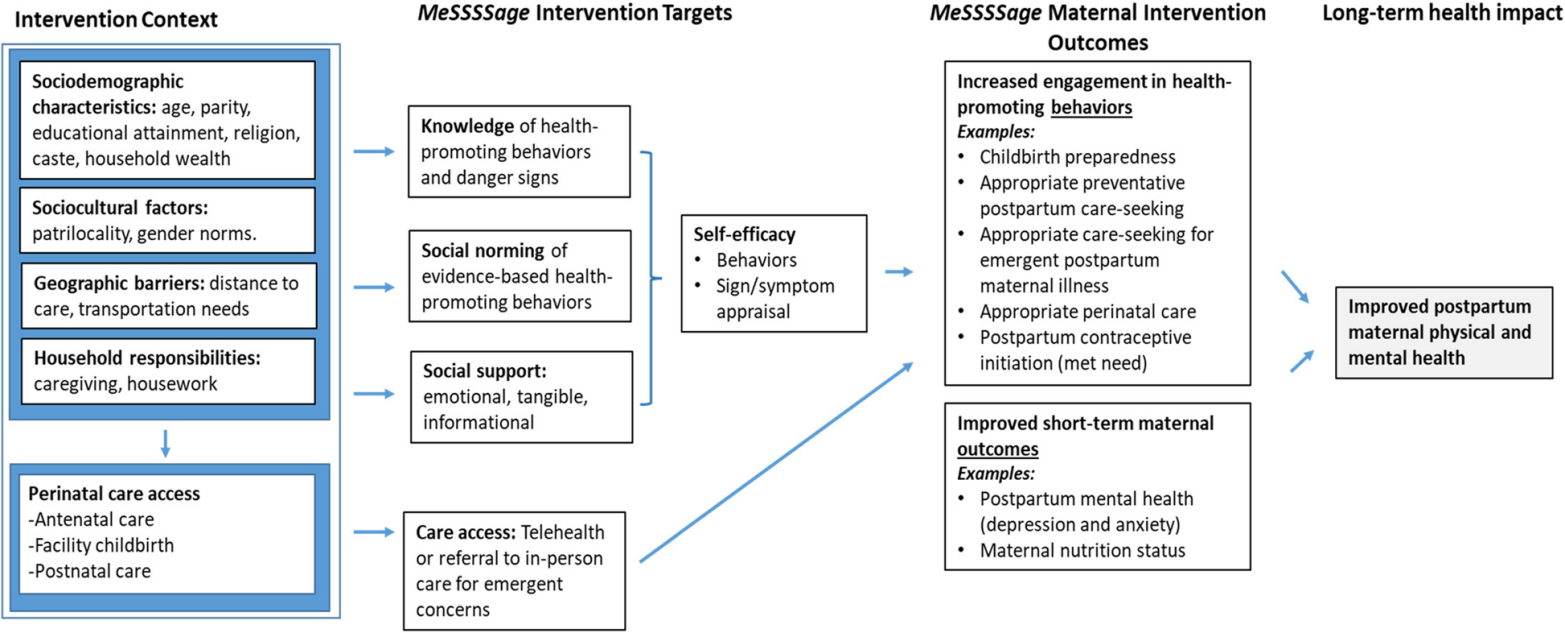
Conceptual Framework of Intervention Context, *MeSSSSage* Intervention Targets, Outcomes and Anticipated Long-Term Impacts. Notes: *MeSSSSage *intervention targets include knowledge, social support, and postnatal care. Primary behavioral and health outcomes are italicized (postpartum depression, exclusive breastfeeding, and postpartum contraceptive uptake)

To address these needs, we developed a group intervention called *MeSSSSage* (Maa Shishu Swasthya Sahayak Samooh meaning maternal and child health support group) to increase perinatal women’s knowledge, refer them to in-person care as needed, and to connect them with a virtual social support group of other mothers with similarly-aged infants through weekly calls and text chat. Informed by the capabilities and motivation constructs of the capabilities, opportunities, motivation and behavior (COM-B) framework [22, 25], the intervention targeted knowledge of health-promoting behaviors and parental self-efficacy and empowerment to impact health knowledge, behaviors, and maternal and child health outcomes (Fig. 1). Our development process included two iterative rounds of pilot testing, the first to inform key platform and design factors [32], and the second to understand feasibility and acceptability of our revised model (Diamond-Smith NG, El Ayadi AM, Duggal M, et al. Feasibility and acceptability of a multi-component mhealth group social support and education intervention for postpartum women in India. Under review.).

In the current analysis, we sought to describe the preliminary effectiveness of the *MeSSSSage m*Health education and social support intervention on maternal health knowledge, behaviors and outcomes at six months postpartum. In this analysis, we focus on changes in maternal health knowledge from pre to post-intervention; postpartum health care seeking and receipt; postpartum physical health, mental health, and functional mobility status; and initiation of postpartum contraception to space or limit births.

## Methods

The *MeSSSSage* mHealth educational and social support intervention pilot study occurred within Boothgarh district, Punjab state, northern India. Eligibility criteria for participation included age 18 or above, 28–32 weeks pregnant, living in the study area, and having no major maternal complications. This open-label pilot study employed a pretest–posttest nonrandomized control group design, with quantitative survey data collected at study enrollment and intervention completion (~ 6 months postpartum) across different combinations of intervention modalities (Table 1).

**Table 1:** *MeSSSSage* intervention modalities

*Intervention Modality*	*Description*
*Audio or video or audio group call sessions (“group call”)*	Trained moderator-led weekly group educational and social support group sessions included icebreakers/group-building activities, facilitated discussions on weekly themes (Fig. 2), and open question/discussion sessions. A gynecologist participated in one call per month prenatally, and a gynecologist and neonatologist participated in one call per month postnatally. Groups were audio only (TATA platform) or video (Zoom platform) per participant preference
WhatsApp-based group text chat	Trained moderator-facilitated WhatsApp group text chats where educational audio and visual messages were shared weekly and group members were encouraged to ask questions for moderator and other group member response
MeSSSage mobile application (app)	Weekly educational audio messages focused on key information regarding weekly themes (Figure X). The mobile app organized sections for weekly audio messages, providing women with the flexibility to access health education content at their convenience
Interactive Voice Response (IVR)	IVR calls were dispatched to participants once per week at designated days and times to share weekly educational audio messages

Our study team pre-screened potential participants using antenatal clinic registry data maintained by community health workers. Potential participants were then contacted over the phone, screened, and if eligible, invited to participate and led through an informed consent process which included a discussion of the study procedures, risks and benefits. Verbal consent was obtained from all participants, and voice recordings of informed consent confirmation were maintained. Of 397 potential participants with whom our study team attempted to contact, we successfully recruited 201 participants. Reasons for non-enrollment included the following: unable to reach the potential participant over cell phone (switched off (*n* = 64), out of service (*n* = 11) or did not pick call (*n* = 17)), wrong number (*n* = 32), no longer pregnant (miscarriage or preterm birth; *n* = 16)), inconsistent access to mobile phone (*n* = 22), not interested (*n* = 25), language barrier (*n* = 2), and not eligible (*n* = 7).

Participant engagement in the intervention lasted a total of eight months; participants were recruited, and the eight groups formed in a staggered fashion, resulting in a study timeline of August 2021 – November 2022. Participant enrollment and baseline quantitative survey administration took place between August -December 2021. Intervention implementation occurred from August 2021 through July 2022, and our endline quantitative survey was administered between May and November 2022. Participant retention across the eight-months of the study was 67% (*n* = 135 participants in endline survey) and ranged from 65%-78% by intervention modality group (78% for group call “synchronous”, 70% in the IVR and WhatsApp “asynchronous” arm, and 65% in the control arm).

### MeSSSage intervention

**Fig. 2:**
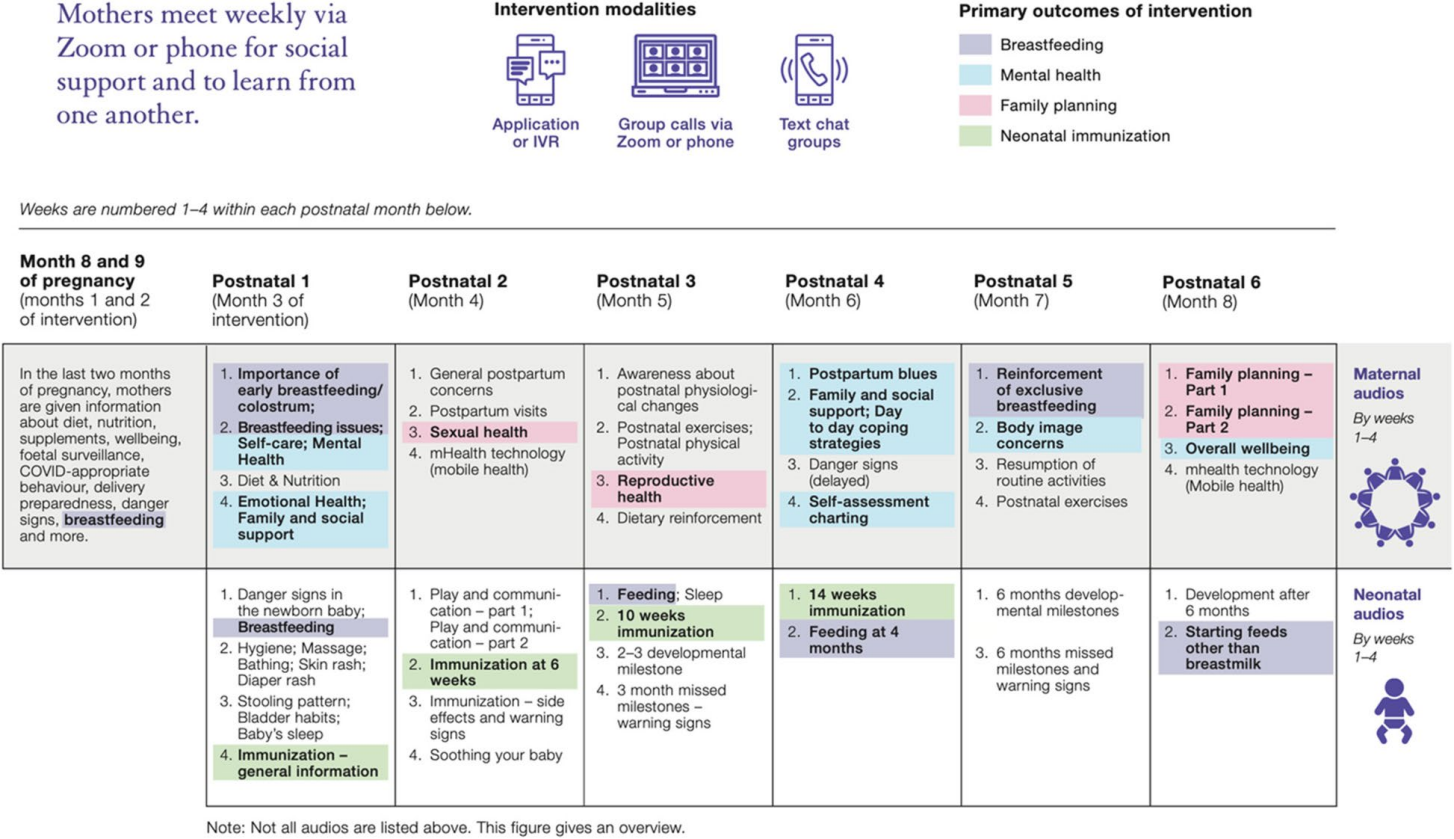
Weekly maternal and neonatal content of *MeSSSage* intervention

A detailed description of the pilot intervention and our assessment of its feasibility and acceptability is provided elsewhere (Diamond-Smith NG, El Ayadi AM, Duggal M, et al. Feasibility and acceptability of a multi-component mhealth group social support and education intervention for postpartum women in India. Under review.); briefly, *MeSSSage* intervention modalities comprised weekly hour-long audio or audio–video group calls, group text chats, and audio educational content provided via automated IVR or application (Table 1 and Fig. 2). Weekly educational content and group calls began prenatally at 32 weeks of gestation (2 sessions) and continued weekly for six months postpartum. Each group included 20 study participants whose gestational ages were within a two-week range of each other to optimize the educational content by specific gestational age. This pilot study assessed eight intervention groups utilizing four different intervention modality combinations: 1) app only (1 group; *n* = 20), 2) IVR only (1 group; *n* = 20), 3) Group call + Whatsapp + App (3 groups, *n* = 60), 4) Group call + Whatsapp + IVR (3 groups, *n* = 60). Groups were not assigned equivalently across the four combinations of intervention modalities due to the pilot nature of the project and our greater interest in learning from synchronous (group call) modality participants. As the group call modality is novel, we preferred to pilot the intervention with multiple groups in these combinations to contribute to a better understanding of intervention implementation. Data were collected from 20 control participants. 21 individuals originally enrolled in the study but withdrew before intervention start and were replaced. If in group call or text chat a physical examination for mother or infant was needed, they were referred to a health provider. All participants, including the control arm, received the standard of care which in this setting, comprised of community health worker-led home visits, counseling, prenatal vitamins, immunization services, and supplemental nutrition services.

For the purposes of analysis, we collapsed study participants into two intervention groups because of our primary interest in understanding the differences in asynchronous (groups 1 and 2 described above) vs. synchronous communication (groups 3 and 4) modalities and intervention engagement. We expected synchronous participants to experience greater social connectedness than those receiving just the educational app or IVR who received asynchronous information without connecting directly with others.

### Study measures

Pre and post-intervention data were collected through interviewer administration over the phone.^1^ Participant sociodemographic characteristics collected at pre-intervention included age, relationship status, educational attainment, religion, caste, ration card and type, parity, and mobile phone ownership. Maternal health knowledge assessed both pre- and post-intervention focused on assessment of nine key maternal health danger signs during pregnancy and childbirth (prolonged labor; excessive bleeding; convulsions; swelling of hands, body or face; high fever; foul-smelling vaginal discharge; severe pain in lower abdomen; uterine water discharge; or other), twelve key maternal health danger signs during postpartum (excessive bleeding; convulsions; swelling of hands, body or face; high fever or severe headache; foul-smelling vaginal discharge; severe pain in lower abdomen; chest pain or shortness of breath; calf pain, redness or swelling; increased perineal pain; problems urinating or leaking; swollen, red or tender breasts or nipples; severe depression or suicidal behavior; or other), and seven key preparations for institutional birth (hospital selection, contact for transportation vehicle, clothes and pads for mother and infant, identify people to accompany mother, save or arrange for money, prepare documents required for birth, identify someone to take care of house during absence, or other). Endline assessment of maternal health behaviors and status focused on postpartum health care use, overall postpartum health and functional mobility, postpartum mental health (i.e., level of depressive and anxiety symptoms, assessed using the Edinburgh Postnatal Depression Scale and the Generalized Anxiety Disorder-7 scale) [33, 34], and current and intended postpartum contraceptive use.

### Ethical approvals

This study received approval from the Health Ministry Screening Committee of the Indian Council of Medical Research and Health Department of Government of Punjab. The study protocol was approved by the University of California, San Francisco Institutional Review Board (19–299,723); the Ethics Committee of the Post Graduate Institute of Medical Education and Research (IEC-03/2020–1567); the Collaborative Research Committee of the Post Graduate Institute of Medical Education and Research (79/30-Edu-13/1089–90); and the Indian Council of Medical Research (ID 2020–9576). Informed consent was obtained by all study participants. All research activities were carried out in compliance with the Helsinki Declaration.

### Data analysis

Given our primary focus on change across time, we limited the analytic sample for this paper to participants with both baseline and endline data (*n* = 135). We first compared the sociodemographic characteristics of the three analysis arms (synchronous, asynchronous (IVR/WhatsApp), and control) through identification of standardized differences [35]. Because we identified significant differences across groups in the distribution of age, age at marriage, household composition, educational attainment, household income and ration card, mobile phone ownership and smart phone access, we employed inverse probability weighting to make participants comparable across arms. This approach is akin to direct standardization and considers multiple differences in underlying demographics between the arms at baseline [36, 37].

We summarized sociodemographic characteristics using proportions and means of the matched, reweighted study population surveyed pre- and post- implementation of the *MeSSSage* intervention stratified by three arms (synchronous, asynchronous (IVR/App), and control). We then assessed the effect of being in each intervention arm (either synchronous, asynchronous, or control arm) on primary outcomes (changes in knowledge of maternal danger signs during pregnancy or postpartum, planning for facility birth planning, and knowledge of family planning methods) using mixed effects linear regression including a random intercept for participant with robust standard errors to adjust within individual clustering due to the longitudinal structure of the data. The difference-in-difference (*Beta*) is the interaction term between a categorical variable denoting the time (before vs. after the intervention was implemented) and the intervention arm (synchronous vs. asynchronous; synchronous vs. control; asynchronous vs. control). We interpreted this term differential change over time in each intervention arm compared to the reference group. For outcomes collected only at endline (postpartum health check, postpartum general health, postpartum depression or anxiety, and postpartum contraceptive method use) we analyzed the differences between the arms (synchronous vs asynchronous intervention, synchronous intervention vs control, and asynchronous intervention vs control) using logistic regression. Differences where *p* < 0.05 were considered statistically significant. All analyses are presented using weighted estimates and were conducted as intent-to-treat. Data entry was done through REDCap, and all statistical analyses were conducted using Stata 15 [38].

## Results

### Sociodemographic characteristics

At enrollment, study participants were mean age 26.8 years (mean 26.8, SD 3.9) and almost all were married (99.3%; Table 2). Most women had either completed a high school education (44.8%) or higher (44.3%). Nearly two-thirds of the sample belonged to the Sikh religion (65.3%) and one-third to marginalized caste (scheduled caste and scheduled tribe; 36.4%). Approximately half of participants (48.0%) possessed a ration card, an official government document given to eligible poor families to get subsidized food grains from government fair price shops. Parity was one (53.3%) or more (46.8%). Mobile phone ownership at the household-level was near-universal (99.2%), and most women owned their own phone (92.5%). Among women with their own phone, 89.7% reported having a smart phone whereas 10.3% reported a feature phone. On average, participants achieved 5.5 antenatal care visits, 65% achieving the Indian national guideline of four or more antenatal care visits (not shown), and most (83%) of participants reported initiating antenatal care in the first trimester of pregnancy.

**Table 2:** Sociodemographic characteristics of *MeSSSSage* intervention participants (*n* = 135)

	Synchronous intervention (*n* = 94)	Asynchronous intervention (*n* = 28)	Control (*n* = 13)	Total (*n* = 135)
Age^a^	26.7 (0.38)	26.8 (0.61)	26.7 (1.57)	26.7 (0.33)
Relationship status				
Married or domestic partnership	94 (100%)	27 (98%)	13 (100%)	134 (99.8%)
Separated	0 (0%)	1 (1.2%)	0 (0%)	1 (0.2%)
Educational attainment				
None	0 (0%)	2 (7.2%)	1 (6%)	3 (1.5%)
Up to secondary	9 (9.6%)	1 (3.1%)	3 (22.2%)	13 (9.4%)
Higher secondary	41 (43.6%)	15 (50.1%)	6 (47.1%)	62 (44.8%)
Diploma or higher	44 (46.8%)	10 (39.6%)	3 (24.7%)	57 (44.3%)
Religion				
Hindu	23 (24.5%)	11 (32.2%)	4 (26.3%)	38 (25.7%)
Muslim	9 (9.6%)	0 (0%)	3 (21.8%)	12 (9%)
Sikh	62 (66%)	17 (67.8%)	6 (51.9%)	85 (65.3%)
Caste				
General	41 (43.6%)	16 (55.7%)	5 (39.9%)	62 (45.2%)
Schedule caste/tribe	36 (38.3%)	6 (21.2%)	6 (48.3%)	48 (36.4%)
Other backward class	15 (16%)	3 (9.8%)	2 (11.8%)	20 (14.8%)
Other	2 (2.1%)	3 (13.3%)	0 (0%)	5 (3.6%)
Ration card				
Yes	45 (47.9%)	16 (45.7%)	5 (35.9%)	66 (48.0%)
No	49 (52.1%)	12 (54.3%)	8 (64.1%)	69 (52.0%)
Parity				
1	50 (53.2%)	14 (51.6%)	8 (60.2%)	72 (53.4%)
> 1	44 (46.8%)	14 (48.4%)	5 (39.8%)	63 (46.6%)
Mobile phone ownership				
Individual	93 (98.9%)	25 (91.6%)	9 (80.6%)	122 (92.5%)
Household	88 (93.6%)	28 (100%)	13 (100%)	134 (99.2%)
Total antenatal care visits^a^	5.3 (4.8–5.7)	6.5 (5.7–7.3)	6.5 (5.1–7.8)	5.5 (5.1–5.9)
Antenatal care initiated first trimester	78 (83.0%)	15 (83.8%)	6 (80.5)	99 (82.9%)

^a^Mean(SD)

### Knowledge of maternal danger signs

Despite increases noted across time, maternal danger signs during pregnancy/at childbirth and in the postpartum period remained relatively low (Table 3). Of eight pregnancy/childbirth and twelve postpartum risk factors, the mean number known ranged from mean 1.13 to 2.05 at baseline and 0.79 to 2.10 at endline across groups (Table 2; Table S1). Assignment to the synchronous intervention was associated with a small but significantly greater increase in the mean number of danger signs known during pregnancy/at childbirth when compared to those in the asynchronous intervention group (mean difference 0.94, 95% CI 0.15–1.73) and those in the control group (mean increase 0.89, 95% CI 0.25–1.52), and in the mean number of postpartum maternal danger signs known (mean increase 0.63, 95% CI 0.02–1.24). No differences were identified between the asynchronous intervention group versus control participants.

**Table 3:** Comparisons between pre and post-intervention maternal health-related knowledge by intervention group (*N* = 135)

Knowledge area	T	Mean (95% CI)			Group*Time Parameter (95% CI)		
		**Synchronous intervention**	**Asynchronous intervention**	**Control**	**Synchronous intervention vs. asynchronous intervention**	**Synchronous intervention vs. control**	**Asynchronous intervention vs. control**
Number of pregnancy/childbirth maternal danger signs known (n = 8)	**BL**	1.13 (1.01, 1.24)	1.79 (1.32, 2.26)	1.23 (0.81, 1.65)	0.94** (0.15, 1.73)	0.89** (0.25, 1.52)	-0.05 (-1.00, 0.90)
	**EL**	2.10 (1.87, 2.33)	1.81 (1.29, 2.35)	1.31 (0.91, 1.71)			
Number of postpartum maternal danger signs known (n = 12)	**BL**	1.87 (1.75, 1.99)	2.05 (1.69, 2.42)	1.92 (1.49, 2.35)	0.26 (-0.40, 0.93)	0.63* (0.02, 1.24)	0.36 (-0.48, 1.21)
	**EL**	1.37 (1.16, 1.59)	1.30 (0.78, 1.80)	0.79 (0.36, 1.23)			
Number of planning for institutional delivery steps known (n = 7)	**BL**	0.89 (0.66, 0.98)	1.01 (0.63, 1.39)	1.20 (0.61, 1.79)	0.48 (-0.17, 1.12)	1.14** (0.46, 1.82)	0.66 (-0.19,—1.52)
	**EL**	2.07 (1.84, 2.31)	1.79 (1.38, 2.20)	1.31 (0.87, 1.76)			
Number of family planning methods known (n = 8)	**BL**	1.93 (1.76, 2.10)	1.71 (1.28, 2.14)	1.39 (1.10, 1.67)	0.51* (0.02, 1.00)	0.47 (-0.24, 1.18)	-0.04 (-0.82, 0.74)
	**EL**	2.36 (2.12, 2.60)	1.63 (1.27, 1.99)	1.34 (0.80, 1.88)			

Time (T): baseline (BL) vs. endline (EL); *** *p* < 0.001, ** *p* < 0.01, * *p* < 0.05; Full model output for these analyses is presented in Table S1

### Knowledge of planning for institutional delivery steps

Knowledge related to planning for institutional delivery (steps known) was similarly low but increased over time (Table 3). Of seven steps total, the mean steps known ranged from 0.89 to 1.20 at baseline and 1.31 to 2.07 at endline. Differences in change over time among synchronous intervention participants were noted only in comparison to the control group, with a statistically significant mean increase of 1.14 (95% CI 0.46–1.82) when compared to the control group; no other group differences were identified.

### Knowledge of family planning methods

Family planning method knowledge was low and increase over time only for synchronous intervention participants (Table 3). Of eight family planning methods, the mean number of methods known at baseline ranged from 1.39 to 1.93 at baseline and 1.34 to 2.36 at endline. Synchronous intervention participants had a statistically significant mean increase of 0.51 (95% CI 0.02–1.00) when compared to those in the asynchronous intervention group.

### Postpartum health care, physical and mental health

Achievement of a maternal postpartum health check with a clinical provider within six weeks of giving birth was reported by half of synchronous participants (50.0%), one-quarter of asynchronous participants (25.7%) and one-fifth of control participants (21.0%; Table 4)). Synchronous participants had nearly three-fold increased odds of postpartum health check compared to asynchronous intervention participants (OR 2.88, 95% CI 1.07–7.74). Differences observed between synchronous intervention and control participants did not meet statistical significance.

**Table 4:** Postpartum health care, physical and mental health, and contraceptive use at endline by intervention group (*N* = 135)

	**Synchronous intervention (*n* = 94)**	**Asynchronous intervention (*n* = 28)**	**Control (*n* = 13)**	**Synchronous intervention vs. asynchronous intervention**	**Synchronous intervention vs. control**	**Asynchronous intervention vs. control**
**Postpartum health care**	n (%)	n (%)	n (%)	OR 95%CI	OR 95%CI	OR 95%CI
Postpartum health check within six weeks	48 (51.6%)	7 (32.4%)	3 (31.9%)	2.22 (0.79, 6.25)	2.27 (0.51, 9.99)	1.02 (0.17, 6.04)
Postpartum health check with a clinical provider	47 (50%)	7 (25.7%)	2 (21.0%)	2.88* (1.07, 7.74)	3.75 (0.74, 18.96)	1.30 (0.20, 8.33)
**Postpartum physical health**						
Experienced postpartum health concern	3 (3.3%)	1 (4.5%)	0 (0%)	0.71 (0.07, 7.52)		
Self-rated health						
Excellent/very good	59 (62.7%)	9 (42.8%)	3 (30%)	2.25 (0.83, 6.09)	3.92 (0.84, 18.22)	1.73 (0.28, 10.46)
Good/Fair/Poor	35 (37.3%)	12 (57.3%)	8 (70%)			
Functional mobility						
High difficulty (More than 1.16)	24 (25.5%)	5 (26.5%)	5 (50%)	0.95 (0.30, 2.97)	0.34 (0.09, 1.36)	0.35 (0.06, 2.03)
Low difficulty (< 1.16 tasks, median)	70 (74.5%)	16 (73.5%)	6 (50%)			
**Postpartum mental health**						
Postpartum depression						
Not likely	51 (54.3%)	17 (59.2%)	8 (58.9%)	1.22 (0.50, 3.00)	1.21 (0.34, 4.31)	0.98 (0.22, 4.35)
Possible/Likely/Probable	43 (45.8%)	10 (40.8%)	5 (41.1%)			
Postpartum anxiety						
Minimal	86 (91.5%)	25 (87.9%)	13 (100%)			
Mild/Moderate/Severe	8 (8.6%)	3 (12.1%)	0 (0%)	0.67 (0.16, 2.87)		
**Postpartum contraception use**						
Currently using contraceptive method^a^				1.27 (0.44, 3.60)	0.26 (0.03, 2.29)	0.20 (0.02, 2.29)
Yes	67 (71.3%)	14 (66.1%)	10 (90.3%)			
No	27 (28.7%)	7 (33.9%)	1 (9.7%)			
Would like to be using a contraceptive method^b^				3.67 (0.34, 39.62)		
Yes	11 (40.7%)	1 (15.8%)	0 (0%)			
No	16 (59.3%)	6 (84.2%)	1 (100%)			
Planning to use contraceptive in future	82 (89.1%)	15 (73.4%)	10 (90.3%)	2.97 (0.90, 9.77)	0.87 (0.09, 8.00)	0.29 (0.03, 3.30)

^***^ *p* < 0.001, ** *p* < 0.01, * *p* < 0.05

^a^among those individuals not pregnant; ^b^ among those individuals not currently using a contraceptive method; c among individuals using a contraceptive method

Few participants experienced postpartum health concerns (3%, 5%, and 0%, respectively across synchronous intervention, other intervention and control groups). Self-rated excellent or very good health varied across groups at 63%, 43% and 30%, respectively across synchronous intervention, asynchronous intervention, and control groups, but these differences were not statistically significant.

No differences across groups were noted in postpartum mental health. The proportion of individuals with potential depressive symptoms was similar across synchronous intervention, asynchronous intervention, and control groups, at 46%, 41%, and 41%, respectively. Some differences were noted in anxiety symptoms which were reported by 9%, 12%, and 0% of respondents, but differences did not meet statistical significance or were untestable.

### Postpartum contraceptive use

The proportion of individuals reporting using a postpartum contraceptive method ranged from 66% in the asynchronous intervention group to 90% in the control group, with no statistically significant differences identified across groups (Table 4). Plan to use contraceptive in future was relatively high, ranging from 73% in asynchronous intervention group to 90% in both synchronous intervention and control groups, and no differences noted across groups.

## Discussion

Our preliminary effectiveness assessment of the *MeSSSSage* mHealth education and social support intervention found our synchronous intervention modality had a positive impact on change in knowledge of maternal danger signs at pregnancy and childbirth, institutional delivery preparatory steps, and family planning methods as well as increased postpartum health check achievement. However, no differences were noted in prevalence of mental health symptoms. While further robust effectiveness testing will be required to determine the true effect of the *MeSSSSage* intervention compared to the standard of care on key maternal and infant health outcomes, these preliminary results are promising, especially in conjunction with confirmation of feasibility and acceptability of *MeSSSSage* (Diamond-Smith NG, El Ayadi AM, Duggal M, et al. Feasibility and acceptability of a multi-component mhealth group social support and education intervention for postpartum women in India. Under review.).

Our findings that *MeSSSSage*’s synchronous educational and social support sessions significantly increased maternal health knowledge are consistent with other literature which has identified that both mHealth interventions [39, 40] and interventions incorporating health within a group self-help context improve knowledge outcomes [41–43]. Social support has been identified as an important mechanism influencing health behaviors within both mHealth interventions and participatory group approaches, incorporates a similar emphasis on development of social support [44]. Our asynchronous intervention modality, which included audio educational messages only, was less effective, and this is similar to other research that identified no difference in knowledge simply through the distribution of written educational materials [45]. These findings and their contextualization provide further support for health education strategies employing social support, and in particular, the potential for integration of mHealth delivery into combined social support and health education interventions.

A major concern identified within this study was the relatively low knowledge of maternal danger signs during pregnancy/childbirth and postpartum, institutional delivery preparedness, and contraceptive methods. While knowledge across domains was higher at follow-up within our synchronous intervention modality, the mean number of danger signs and institutional delivery preparatory steps remained only at two and family planning methods just over one. This low level of knowledge is inconsistent with most study participants having initiated antenatal care in the first trimester and met Indian national guidelines for achievement of four or more antenatal care visits. However, other research on levels of maternal health knowledge in India supports low knowledge and educational quality on maternal danger signs even in the context of antenatal care [46–48]; however, results are mixed [49]. Studies have suggested knowledge deficits despite adequate care access may be due to quality concerns, including limited time with providers and lack of formal antenatal education classes, among others [46]. Equipping front-line health workers with mHealth tools has also been found to increase quality of counseling when compared to standard care [27].

The largest impact associated with the synchronous intervention compared to asynchronous intervention participants was the three-fold increased odds of postpartum health check with a clinical provider. Given significant drops in the perinatal continuum of care achievement occurring postpartum for Indian women [50, 51], and the important role of postpartum care in optimizing maternal health and well-being [13, 52], this is an encouraging and finding and may be a way to combat the low rates of postpartum care visits for Indian women. Future research should explore the timing and mechanism of this change, including the timing of increased postpartum visits, and mechanism of impact (e.g., whether this was due to increased knowledge about the importance of postpartum visits, changes in social norms, pressure from group dynamics, or increased accessibility).

Our preliminary effectiveness findings identified no impact of the synchronous intervention modality on postpartum mental health status. We had hypothesized that this modality would have a mental health impact given the strong relationship between social support and maternal mental health [53] and the relatively high prevalence of postpartum depression reported in India [54]. Further inquiry into the intervention impact on mental health will be integrated into subsequent research where more robust research design and sample size will allow for assessment of effectiveness and potential mechanisms of impact.

In combination with our team’s findings confirming the feasibility and acceptability of multiple combinations of *MeSSSSage* modalities, these findings support preliminary effectiveness of this mHealth education and social support intervention (Diamond-Smith NG, El Ayadi AM, Duggal M, et al. Feasibility and acceptability of a multi-component mhealth group social support and education intervention for postpartum women in India. Under review.). However, since our study was designed for feasibility and acceptability assessment, a number of limitations exist relating to estimation of preliminary effectiveness. We combined multiple group modalities to estimate the impact of synchronous versus asynchronous intervention engagement and control, and due to our interest in understanding the potential social support and implementation considerations of a synchronous intervention, we selected to make this our largest group. Our sample size was relatively small and characterized by baseline imbalance across groups in participant sociodemographic characteristics. Weighting was employed to overcome this imbalance; however, future research using more appropriate study designs and sample sizes for estimating intervention effectiveness will provide more robust conclusions. Given the ongoing importance of social and structural factors in perinatal health care continuity in India [55], subgroup evaluation for identifying interventions capable of reducing health inequity will be important.

## Conclusion

Expansion of the evidence base for interventions to improve postpartum care and support are needed to overcome deficits in postpartum care seen across multiple settings. MHealth interventions such as *MeSSSSage* which combine education, social support, and referral may be an important strategy for efficiently reaching this target population, particularly in locations where mobile penetration is high, as a supplement to strategic health systems improvements. Continued advancements in supportive care models may broadly contribute to reducing maternal and neonatal mortality through increasing knowledge and supporting health-promoting behaviors.

## Supplementary Information

Supplementary Material 1.

## Data Availability

No datasets were generated or analysed during the current study.

## Footnotes

1 For individuals who were not able to be reached for endline survey, local ASHAs were engaged to facilitate in-person quantitative survey administration.
