# Supplementary material for: Preliminary impact of an mHealth education and social support intervention on maternal health knowledge and outcomes among postpartum mothers in Punjab, India
Source: BMC Pregnancy Childbirth. 2025 Mar 5;25:239. doi: 10.1186/s12884-025-07310-y (PMC11883990; doi:10.1186/s12884-025-07310-y)
Supplement: Supplementary file 1 — Supplementary Material 1. [file 12884_2025_7310_MOESM1_ESM.docx]

**Supplementary Tables and Figures**

**Table S1. Comparisons between pre and post-intervention maternal health-related knowledge by intervention group (N=135)**

|  | **Group call vs other** | | | **Group call vs control** | | | **Other vs control** | | |
| --- | --- | --- | --- | --- | --- | --- | --- | --- | --- |
| **Number known** | **Group β** | **Time β** | **G*T β** | **Group β** | **Time β** | **G*T β** | **Group β** | **Time β** | **G*T β** |
|  | (95% CI) | (95% CI) | (95% CI) | (95% CI) | (95% CI) | (95% CI) | (95% CI) | (95% CI) | (95% CI) |
| Pregnancy/childbirth maternal danger signs^a^ | -0.66** | 0.03 | 0.94** | -0.10 | 0.07 | 0.89** | 0.56 | 0.07 | -0.05 |
|  | (-1.14, -0.18) | (-0.71, - 0.77) | (0.15, 1.73) | (-0.53, - 0.33) | (-0.49, - 0.65) | (0.25, 1.52) | (-0.07 - 1.19) | (-0.50 - 0.65) | (-1.00, 0.90) |
| Postpartum maternal danger signs^a^ | -0.18 | -0.76* | 0.26 | -0.05 | -1.13*** | 0.63* | 0.13 | -1.12*** | 0.36 |
|  | (-0.56, 0.20) | (-1.38, -0.14) | (-0.40, 0.93) | (-0.49 - 0.40) | (-1.70, -0.56) | (0.02, 1.24) | (-0.43 - 0.70) | (-1.70, -0.55) | (-0.48, 1.21) |
| Institutional delivery steps^a^ | -0.19 | 0.78** | 0.48 | -0.38 | 0.11 | 1.14** | -0.19 | 0.11 | 0.66 |
|  | (-0.60, 0.22) | (0.19, 1.36) | (-0.17, 1.12) | (-0.99 - 0.22) | (-0.50, - 0.73) | (0.46, 1.82) | (-0.89 - 0.51) | (-0.51, - 0.74) | (-0.19, - 1.52) |
| Family planning methods^a^ | 0.23 | -0.08 | 0.51* | 0.00 | -0.04 | 0.47 | 0.32 | -0.04 | -0.04 |
|  | (-0.22, 0.70) | (-0.50, 0.33) | (0.02, 1.00) | (0.21, 0.88) | (-0.7 - 0.61) | (-0.24, 1.18) | (-0.19. 0.84) | (-0.70 - 0.62) | (-0.82, 0.74) |

**** p<0.001, ** p<0.01, * p<0.05; ^a^mean(sd); Group (G); Time (T)*

**Table S2. Postpartum health care, physical and mental health, and contraceptive use at endline by intervention group (N=135)**

|  | **Group call (n=94)** | **Other (n=28)** | **Control (n=13)** |
| --- | --- | --- | --- |
| **Postpartum health care** | n (%) | n (%) | n (%) |
| Postpartum health check within six weeks | 48 (51.6%) | 7 (32.4%) | 3 (31.9%) |
| Postnatal health check with a clinical provider | 47 (50%) | 7 (25.7%) | 2 (21%) |
| **Postpartum physical health** |  |  |  |
| Experienced postpartum health concern | 3 (3.3%) | 1 (4.5%) | 0 (0%) |
| Self-rated health |  |  |  |
| Excellent | 24 (25.5%) | 2 (9%) | 0 (0%) |
| Very good | 35 (37.2%) | 7 (33.8%) | 3 (30%) |
| Good | 26 (27.7%) | 8 (40.7%) | 8 (70%) |
| Fair | 8 (8.5%) | 3 (12.1%) | 0 (0%) |
| Poor | 1 (1.1%) | 1 (4.5%) | 0 (0%) |
| Functional mobility |  |  |  |
| High difficulty (More than 1.16) | 24 (25.5%) | 5 (26.5%) | 5 (50%) |
| Low difficulty (<1.16 tasks, median) | 70 (74.5%) | 16 (73.5%) | 6 (50%) |
| **Postpartum mental health** |  |  |  |
| Postpartum depression |  |  |  |
| Not likely | 51 (54.3%) | 17 (59.2%) | 8 (58.9%) |
| Possible | 41 (43.6%) | 9 (32%) | 5 (41.1%) |
| Likely | 1 (1.1%) | 0 (0%) | 0 (0%) |
| Probable | 1 (1.1%) | 2 (8.8%) | 0 (0%) |
| Postpartum anxiety |  |  |  |
| Minimal | 86 (91.5%) | 25 (87.9%) | 13 (100%) |
| Mild | 7 (7.5%) | 3 (12.1%) | 0 (0%) |
| Moderate | 1 (1.1%) | 0 (0%) | 0 (0%) |
| Severe |  |  |  |
| **Postpartum contraception use** |  |  |  |
| Currently using contraceptive method^a^ |  |  |  |
| Yes | 67 (71.3%) | 14 (66.1%) | 10 (90.3%) |
| No | 27 (28.7%) | 7 (33.9%) | 1 (9.7%) |
| Would like to be using a contraceptive method ^b^ |  |  |  |
| Yes | 11 (40.7%) | 1 (15.8%) | 0 (0%) |
| No | 16 (59.3%) | 6 (84.2%) | 1 (100%) |
| Planning to use contraceptive in future | 82 (89.1%) | 15 (73.4%) | 10 (90.3%) |

*Notes: ^a^ among those individuals not pregnant; ^b^ among those individuals not currently using a contraceptive method; c among individuals using a contraceptive method.*
